# Distinct dementia atrophy progression patterns associated with different epidemiological risk factors

**DOI:** 10.1002/alz.088188

**Published:** 2025-01-09

**Authors:** Alexander David Shapson‐Coe, Sheena Waters, Neil P Oxtoby, Charles R Marshall

**Affiliations:** ^1^ Queen Mary, University of London, London, London UK; ^2^ Queen Mary University of London, London UK; ^3^ University College London, London UK

## Abstract

**Background:**

Most evidence about early structural brain change in dementia derives from highly selected disease cohorts. However, at population level, dementia is typically due to mixed pathologies. We aimed to characterise distinct patterns of brain volume change in early all‐cause dementia in a population cohort.

**Method:**

We used the Subtype and Stage Inference (SuStaIn) algorithm to identify distinct subtypes of disease progression based on structural MRI in the UK Biobank cohort of 152 dementia cases, of which 31 were prevalent cases at the time of scanning and 121 were subsequent incident cases that had been scanned during the pre‐diagnosis phase. We compared other characteristics between subtypes to derive insights into potential aetiologies.

**Result:**

Two distinct subtypes of progression were identified by SuStaIn, one that replicated the typical anatomy of Alzheimer’s disease (AD) with early atrophy of hippocampi and temporal cortex (n=82), and a less common subtype (n=34) characterised by early atrophy of the cerebellum, hippocampus and other subcortical structures, and likely to represent mixed AD and vascular pathologies. Individuals with the latter subtype had significantly higher levels of biomarkers associated with vascular disease, including body mass index (p=0.025) and burden of peri‐ventricular white matter hyperintensities (p=0.020). There was no difference in polygenic risk for Alzheimer’s disease or ApoE4 allele frequency between subtypes.

**Conclusion:**

Subtype and Stage Inference provides a data‐driven approach to identifying distinct patterns of dementia progression. Within the UK Biobank cohort of dementia cases, these subtypes appear to be associated with distinct biological risk factors, which may improve our understanding of the earliest stages of dementia.

